# Genetic polymorphisms in the renin-angiotensin system and cognitive decline in Parkinson’s disease

**DOI:** 10.1007/s11033-021-06569-6

**Published:** 2021-07-23

**Authors:** Anna Pierzchlińska, Jarosław Sławek, Monika Mak, Barbara Gawrońska-Szklarz, Monika Białecka

**Affiliations:** 1 Department of Pharmacokinetics and Therapeutic Drug Monitoring, Pomeranian Medical University, Powstańców Wlkp 72, 70-111 Szczecin, Poland; 2 Department of Neurological-Psychiatric Nursing, Faculty of Health Sciences, Medical University of Gdansk, Gdansk, Poland; 3 Department of Neurology, St Adalbert Hospital, Gdansk, Poland; 4 Department of Health Psychology, Pomeranian Medical University, Szczecin, Poland

**Keywords:** Parkinson’s disease, Renin-angiotensin system, Dementia, Cognitive decline, Genetic polymorphisms

## Abstract

**Background:**

Renin-angiotensin system (RAS) influences the central nervous system not only through its peripheral impact—the brain possesses its own local RAS. Studies showed altered RAS components in Parkinson’s disease (PD) and their association with oxidative stress which may be linked to neurodegeneration and dementia. Moreover, the protective functions of RAS blockade antagonists against cognitive decline and dementia have been suggested. This study aimed to examine whether genetic variability in RAS genes correlates with cognitive decline in PD.

**Methods and results:**

We genotyped single nucleotide polymorphisms (SNPs) in angiotensinogen (*AGT*: rs699, rs4762), angiotensin II receptors (*AGTR1*: rs5186 and *AGTR2*: rs5194, rs1403543) genes, as well as insertion/deletion polymorphism in the angiotensin-converting enzyme (*ACE* I/D) gene in 256 PD patients, divided into three groups: without cognitive decline, with mild cognitive impairment and with PD dementia. We did not find any significant differences in the frequencies of the analysed polymorphisms in any of the groups.

**Conclusions:**

Despite no direct correlation between the investigated polymorphisms in RAS genes and cognitive decline in PD, we believe the impact of those genotypes may be indirect, affecting RAS blockade treatment.

**Supplementary Information:**

The online version contains supplementary material available at 10.1007/s11033-021-06569-6.

## Introduction

The impact of the renin-angiotensin system (RAS) on the central nervous system was originally considered by its influence on blood pressure and water and electrolyte balance since RAS components do not cross the blood–brain barrier [1]. Angiotensin-converting enzyme (ACE) forms the main effector peptide of RAS—angiotensin II (AII), which acts via two receptors: AT1 (AT1R) or AT2 (AT2R) [2]. Further research confirmed that RAS exists also locally in many tissues, including the brain where astrocytes produce the precursor peptide to AII – angiotensinogen [3, 4].

Some components of RAS have been found to be altered in Parkinson’s disease (PD) patients: ACE activity was increased in the cerebrospinal fluid and AT1R expression was decreased in the brain in a *post-mortem* study, which was associated with the loss of dopaminergic neurons [5, 6]. Villar-Cheda et al. [2] presented a correlation between AT1R expression levels and dopamine levels in rodents—the expression was higher in D1 or D2 dopamine receptor-deficient mice than in the controls. Angiotensin II activates the NADPH oxidase complex via AT1R which leads to reactive oxygen species (ROS) generation, causing oxidative stress and cell apoptosis as a consequence, including dopaminergic neurons [7, 8]. The induction of parkinsonian symptoms was observed in PD animal models by the use of a neurotoxin that increased NADPH expression and microglia activation; moreover, increased expression of NADPH:quinone oxidoreductase was observed in the substantia nigra *pars compacta* of PD patients, among whom many were described as having dementia [8, 9]. In the in vitro studies, ROS formation was diminished by AT1R antagonists or NADPH oxidase inhibitor [7]. However, AT2R antagonists acted in the opposite way and AT2R agonists exerted neuroprotective functions [10].

Therapy with ACE inhibitors (i.e. captopril, perindopril) exerted neuroprotective effects in a PD animal model; moreover, PD patients on perindopril reacted faster on their levodopa (l-dopa) medication, had reduced dyskinesia, and were more active during the day [11, 12]. Several studies showed protective functions of RAS blockade—by ACE inhibitors or AT1R antagonists—against cognitive decline and dementia (in non-PD patients), or the progression of PD [13–15]. The severity of PD has been correlated with dementia [16, 17], thus slowing the progression of motor symptoms may be a protective factor against cognitive decline.

The prevalence of dementia in Parkinson’s disease (PDD) is very high, as it can affect up to 80% after 12 years, significantly decreasing patients’ quality of life and leading to their disability [18]. The aetiology of cognitive decline in PD is unclear and multifactorial; some susceptibility factors have been established: e.g. higher age, fewer years of education, longer disease duration, higher age-at-onset, higher l-dopa dose, more severe autonomic and depressive symptoms, as well as genetic susceptibility factors [16, 19]. The impact of the genetic variability in RAS on PD has been analysed [20]; however, no studies on its influence on cognitive decline in PD have been performed.

The aim of the presented research was to establish the impact of several polymorphisms in RAS—in the genes of angiotensinogen, ACE, AT1R, and AT2R—on the risk of mild cognitive impairment (MCI) or dementia in PD.

## Methods

### Subjects

The study population comprised of 256 patients of Caucasian origin (116 males and 140 females), aged from 35 to 89 (64.5 ± 10.0), from two urban centres in Poland (Gdansk, Szczecin). The subjects were diagnosed with idiopathic PD according to UK Parkinson’s Disease Society Brain Bank clinical diagnostic criteria [21]. All patients with clinical symptoms suggesting secondary causes of the parkinsonian syndrome (vascular, drug-induced), with features suggestive of atypical parkinsonian syndromes (multiple system atrophy, progressive supranuclear palsy and corticobasal syndrome) or with the presence of cardiovascular disease (e.g. stroke, heart failure) were excluded from final data analysis. Informed written consent was obtained before participation. The protocol of the study was approved by the relevant local ethics committee.

Based on the neuropsychological assessment described below, the group was divided into three subgroups: PD patients without MCI or dementia (PD-non cognitive impairment, PD-NCI, n = 68), PD patients with MCI (PD-MCI = 122), and PD patients with dementia (PDD, n = 66). Demographic and clinical data were collected according to a semi-structured interview and medical documentation and are presented in Table 1.

**Table 1:** Demographic and clinical characteristics of PD patients without cognitive impairment (PD-NCI), with mild cognitive impairment (PD-MCI) and patients with Parkinson’s disease dementia (PDD)

Demographic and clinical data	PD-NCI patients (n = 68)	PD-MCI patients (n = 122)	PDD patients (n = 66)	p value
Males/females	42/26	62/60	36/30	0.348^a^
Age [years] mean, SD, range	63.3 ± 10.6 43–89 (n = 68)	62.7 ± 9.4 39–87 (n = 122)	69.0 ± 9.1 35–85 (n = 66)	< 0.001^b^
Age at disease onset [years] mean, SD, range	57.6 ± 11.1 37–87 (n = 68)	56.4 ± 11.0 28–80 (n = 122)	60.3 ± 10.5 29–77 (n = 66)	0.017^b^
Disease duration [years] mean, SD, range	5.7 ± 4.4 0.5–21 (n = 68)	6.3 ± 4.9 1–21 (n = 122)	8.6 ± 5.9 0.5–24 (n = 66)	0.034^c^
MMSE mean, SD, range	28.8 ± 1.4 25–30 (n = 43)	28.1 ± 1.7 24–30 (n = 118)	23.5 ± 4.3 11–30 (n = 65)	< 0.001^c^
UPDRS (part II-IV) score mean, SD, range	24.5 ± 11.5 1–54 (n = 59)	30.7 ± 15.7 7–80 (n = 111)	46.8 ± 21.0 6–101 (n = 61)	< 0.001^c^
Daily l-dopa dosage [mg]	606.2 ± 337.6 150–1750 (n = 63)	722.8 ± 447.5 150–1900 (n = 118)	820.9 ± 436.5 100–2000 (n = 64)	< 0.001^c^

p values calculated by means of: ^a^χ^2^ test; ^b^one-way parametric ANOVA test; ^c^one-way non-parametric ANOVA test (Kruskal–Wallis test)

### Neurological examination

Neurological examination was performed to confirm the PD diagnosis and exclude other symptoms suggesting atypical or symptomatic cases. It consisted of the Unified Parkinson’s Disease Rating Scale (UPDRS; part II–IV), Hoehn–Yahr staging, and the Schwab-England activities of daily living scale. It was followed by magnetic resonance imaging to exclude other aetiologies.

### Neuropsychological assessment

All assessments were conducted by an experienced psychologist, who established examination procedures and their standards before the onset of the study. Patients were examined in the ‘on state’. The Mini-Mental State Examination (MMSE) test was used as a screening tool. Detailed neuropsychological examination, including the Wechsler Adult Intelligence Scale-Revised (WAIS-R), the Rey Auditory Verbal Learning Test (RAVLT), the Benton Visual Retention Test (BVRT), the Trail Making Test (TMT), the Rey-Osterrieth Complex Figure Test (ROCF), the Verbal Fluency Test and the Wisconsin Card Sorting Test (WCST) was performed. The Beck Depression Inventory Test (BDI) was used to assess mood disturbances. In addition, all patients were examined by means of Parkinson’s Disease—Cognitive Rating Scale (PDCRS). The diagnosis of dementia was established in accordance with Emre et al.’s criteria [22].

### Genetic study

Peripheral venous blood samples were collected from each subject into tubes containing EDTA. Then extraction of genomic DNA using a Genomic Mini AX Blood SPIN was performed (A&A Biotechnology, Poland). The concentration of every sample of DNA was measured spectrophotometrically by Nanodrop ND-1000 (Thermo Scientific, USA) and diluted to 20 ng/ml. To determine polymorphisms in the genes of angiotensinogen (*AGT*: rs699, rs4762), AT1R (*AGTR1*: rs5186) and AT2R (*AGTR2*: rs5194, rs1403543) real-time PCR using pre-validated allelic discrimination TaqMan assays (rs699 assay ID: C_1985481_20, rs4762 assay ID: C_1985480_20, rs5186 assay ID: C_3187716_10, rs5194 assay ID: C_1841567_20, rs1403543 assay ID: C_7481825_10; Thermo Scientific, USA) was carried out. The insertion/deletion (I/D) polymorphism in the angiotensin-converting enzyme’s gene (*ACE*) was analysed by PCR with the second round of amplification for D homozygous carriers due to preferential amplification of D allele [23]. The genotypes of *ACE* I/D were determined by running the products in 3% agarose gel with ethidium bromide (1 μg/mL) for UV visualization. The buffer used was 1X Tris–borate-EDTA (TBE) buffer (Thermo Scientific, USA).

### Statistical analysis

Concordance of genotypes distributions with Hardy–Weinberg equilibrium was assessed using the χ^2^ test (for X-linked gene—*AGTR2*—only in women). Genetic case–control analyses between study groups were performed using the χ^2^ test (between 3 groups) or Fisher exact test (between 2 groups). The minor alleles of single nucleotide polymorphisms were chosen according to the ALFA Allele Frequency database (https://www.ncbi.nlm.nih.gov/snp/docs/gsr/alfa/) for the European population, the minor allele of *ACE* insertion/deletion was chosen according to the available literature. Odds ratios (OR) and 95% confidence intervals (95% CI) were calculated using Wald’s method with the continuity correction. The polymorphisms located on the X chromosome (*AGTR2* rs5194 and rs1403543) were also presented separately for men and women. For demographic and clinical data, the alignment with normal distribution was tested by means of the Shapiro–Wilk test, and further analyses were performed by means of a one-way parametric ANOVA test or one-way non-parametric ANOVA test (Kruskal–Wallis test). A p value of less than 0.05 was considered statistically significant. The analyses were performed in Statistica ver. 13.2 (TIBCO Software Inc., USA).

## Results

The groups of PD-NCI, PD-MCI, and PDD did not differ in terms of sex, but varied significantly in the mean age of the participants, disease duration, age at disease onset, MMSE score, UPDRS score, and daily l-dopa dosage, with the greatest mean values for all of them in PDD group (Table 1).

The genotype distributions of all tested polymorphisms, except for *ACE* I/D in PD-MCI group, were in Hardy–Weinberg equilibrium (Table 2). The number of *ACE* heterozygous carriers in PD-MCI was expected to be lower (n = 60, 50.0%), while both the numbers of the homozygous carriers were expected to be higher (DD n = 28.5, 23.75%; II n = 31.5, 26.25%), according to the χ^2^ test calculation.

**Table 2:** Frequencies of the studied polymorphisms in PD patients without cognitive impairment (PD-NCI), with mild cognitive impairment (PD-MCI) or patients with Parkinson’s disease dementia (PDD)

Polymorphism	Genotype/allele	PD-NCI n (%)	PD-MCI n (%)	PDD n (%)	p value
*AGT* rs699:T > C	TT	15 (22.1)	33 (27.3)	20 (30.8)	0.535
	CT	33 (48.5)	63 (52.1)	28 (43.1)	
	CC	20 (29.4)	25 (20.7)	17 (26.1)	
	CT + CC	53	88	45	0.518
	Minor allele (C) frequency	(53.7)	(46.7)	(47.7)	0.409
*AGT* rs4762:C > T	CC	45 (66.2)	86 (71.1)	51 (78.5)	0.560
	CT	20 (29.4)	32 (26.4)	13 (20.0)	
	TT	3 (4.4)	3 (2.5)	1 (1.5)	
	CT + TT	23	35	14	0.285
	Minor allele (T) frequency	(19.1)	(15.7)	(11.5)	0.233
*AGTR1* rs5186:A > C	AA	37 (54.4)	60 (49.6)	35 (53.8)	0.886
	AC	27 (39.7)	52 (43.0)	24 (37.0)	
	CC	4 (5.9)	9 (7.4)	6 (9.2)	
	AC + CC	31	61	30	0.767
	Minor allele (C) frequency	(25.7)	(29.0)	(27.7)	0.802
*AGTR2* (X chr.) rs5194:A > G	G	47 (50.5)	81 (45.3)	46 (49.5)	0.750
	A	46 (49.5)	98 (54.7)	47 (50.5)	
	Minor allele (A) frequency	(49.5)	(54.7)	(50.5)	0.750
*AGTR2* (X chr.) rs5194:A > G only men	G	21 (51.2)	30 (49.2)	20 (57.1)	0.751
	A	20 (48.8)	31 (50.8)	15 (42.9)	
	Minor allele (A) frequency	(48.8)	(50.8)	(42.9)	0.751
*AGTR2* (X chr.) rs5194:A > G only women	GG	5 (19.2)	10 (16.9)	5 (17.2)	0.884
	AG	16 (61.5)	31 (52.5)	16 (55.2)	
	AA	5 (19.2)	18 (30.5)	8 (27.6)	
	AG + AA	21	49	24	0.967
	Minor allele (A) frequency	(50.0)	(56.8)	(55.2)	0.714
*AGTR2* (X chr.) rs1403543:G > A	A	46 (49.5)	90 (50.3)	47 (50.5)	0.988
	G	47 (50.5)	89 (49.7)	46 (49.5)	
	Minor allele (G) frequency	(50.5)	(49.7)	(49.5)	0.988
*AGTR2* (X chr.) rs1403543:G > A only men	A	21 (51.2)	28 (45.9)	19 (54.3)	0.710
	G	20 (48.8)	33 (54.1)	16 (45.7)	
	Minor allele (G) frequency	(48.8)	(54.1)	(45.7)	0.710
*AGTR2* (X chr.) rs1403543:G > A only women	AA	5 (19.2)	16 (27.1)	6 (20.7)	0.933
	AG	15 (57.7)	30 (50.8)	16 (55.2)	
	GG	6 (23.1)	13 (22. 0)	7 (24.1)	
	AG + GG	21	43	23	0.666
	Minor allele (G) frequency	(51.9)	(47.5)	(51.7)	0.805
*ACE* I/D	DD	9 (13.4)	21 (17.5)^a^	10 (15.4)	0.662
	ID	39 (58.2)	75 (62.5)^a^	37 (56.9)	
	II	19 (28.4)	24 (20.0)^a^	18 (27.7)	
	ID + II	58	99	55	0.760
	Minor allele (I) frequency	(57.5)	(51.3)	(56.2)	0.446

p values for alleles and genotypes calculated by means of χ^2^ test in relation to major allele or homozygotes for a major allele

^a^the genotype distribution not in Hardy–Weinberg equilibrium

The genotype and allele frequencies of any of the analysed polymorphisms did not vary significantly between the groups. The highest observed difference was noted for the minor allele of *AGT* rs4762:C > T polymorphism; however, without statistical significance. Minor alleles, whose frequencies are reported to be around 50%, in some cases, were more often observed than the major alleles, i.e. *AGT* rs699:C in PD-NCI, *AGTR2* rs5194:A in PD-MCI (men and women) and PDD (women), *AGTR2* rs1403543:G in PD-MCI (men), PD-NCI (women), PDD (women), *ACE* I/D in all three groups.

The comparisons of the genotypes and alleles distributions between the groups: (1) PD-NCI vs. PD-MCI + PDD, (2) PD-NCI + PD-MCI vs. PDD, (3) PD-NCI vs. PD-MCI, or (4) PD-NCI vs. PDD did not reveal any significant differences (Supplementary Table 1).

## Discussion

We have found that none of the analysed polymorphisms in the genes of renin-angiotensin system, i.e. rs699, rs4762 in *AGT*, rs5186 in *AGTR1*, rs5194, rs1403543 in *AGTR2* or *ACE* I/D was associated with MCI or dementia in Parkinson’s disease.

Up to date, no study has investigated the correlation between the genetic variability in RAS genes and cognitive impairment in PD. The most widely studied of the mentioned genetic variants in the context of PD or cognitive impairment was the *ACE* I/D polymorphism which is responsible for a significant variance in ACE levels—the DD genotype results in two-fold higher plasma and tissue ACE levels than in the II carriers [24]. Song and Lee [20] in their meta-analysis, including five studies on PD, did not find any association between the *ACE* I/D polymorphism and PD risk, and none of the included studies reported a correlation between *ACE* I/D and PD either. There was no information on the cognitive status of the participants. However, the analysis revealed an observation about the frequency of the polymorphism among different ethnic groups—in the control groups, PD group and schizophrenia group, D allele frequency was around 53% in Europeans and Australians, but only 29% in the Turkish population, and 34% in Asians. Huo et al. [25] did not find an impact of *ACE* I/D polymorphism on the occurrence of PD either (Asian population). Since ACE activity was elevated in the cerebrospinal fluid of PD patients longitudinally treated with l-dopa [5], the polymorphism was analysed in association with the side effects of the drug. The studies did not find a correlation with dyskinesia or fluctuations [26, 27], but Lin et al. [26] reported higher risk of l-dopa-induced psychosis among II homozygotes (OR 2.542, 95% CI 0.034–0.242, p = 0.012).

The impact of RAS genes variability on dementia, Alzheimer’s disease (AD) and vascular dementia was analysed in two Swedish studies [28, 29]. One of them reported a two-fold higher risk of dementia in *ACE* II homozygotes compared to combined ID and DD genotypes (OR 2.17; 95% CI 1.22–3.85; p = 0.008); moreover, II carriers were more likely to develop dementia under the age of 70 (OR 4.35; 95% CI 1.37–13.86; p = 0.013) [28]. In a subsequent study, the authors revealed no correlation between the *ACE* I/D polymorphism and dementia during the follow-up, likewise with *AGTR1* rs5186, whose CC genotype was associated with dementia only at baseline (OR 3.25; 95% CI 1.42–7.06; p = 0.001) [29]. Unfortunately, the results were not presented for AD and vascular dementia separately. There was also no information about the inclusion of PDD in the analysis. Dementia is a complex term, with various pathogenesis and clinical presentation, thus susceptibility factors, including genetic polymorphisms, may lack resemblance in different types of dementia. Such an example is a variant of the apolipoprotein E gene—*APOE4*—which is an established risk factor for AD; whereas, studies on PDD showed conflicting results [30].

We assume that polymorphisms in RAS genes may exert an indirect influence on cognitive decline in PD. Some studies revealed a correlation between white matter hyperintensities (WMH) seen in magnetic resonance imaging with dementia in PD [31–33]. Taylor et al. [34] analysed polymorphisms in both angiotensin II receptors genes: A1166C (rs5186) in *AGTR1* and C3123A (rs2148582) in *AGTR2*. Although no correlation was found between the polymorphisms and WMH in women, WMH volume changed less in male 1166A homozygotes compared to other A1166C genotypes. The authors also showed a protective impact of *AGTR2* 3123C allele against WMH volume change in hypertensive men. However, the results were not supported by Gebril et al. [35], as they found no association between *AGTR1* A1166C and WMH development in the aging brain. A polymorphism in *AGT* gene (rs699) was reported to correlate not only with the white matter integrity, but also with cognition in healthy adults—mutant TT homozygotes performed worse in tests assessing attention, processing speed and language functions [36].

Another indirect influence of RAS genes variability on cognitive functions, also in PD patients, may be that it alters the neuroprotective benefits of ACE inhibitors. Hajjar et al. conducted a study with 8-year’s follow-up on 3000 cognitively intact elderly participants—Caucasians and African American, of whom 15% were treated with ACE inhibitors [37]. They found that the medication was protective against cognitive decline in AA carriers of *AGT* 6AG (rs5051), and in CC homozygotes of the *AGT* M235T (rs699) polymorphism, both associated with higher angiotensinogen levels. Therefore, only in those carriers the inhibitors may show neuroprotective effect by decreasing RAS activity. The association was significant only in Caucasians; moreover, no impact of the *ACE* I/D polymorphism was found in either group.

We describe here the classical RAS pathway and polymorphisms within its genes. A rising number of publications has focused on the alternative axis, i.e., angiotensin converting enzyme 2 (ACE2), angiotensin (1-7) [Ang-(1-7)] produced by ACE2 from AII, and Mas receptor. Activating the ACE2/Ang-(1-7)/Mas axis in animal models resulted in neuroprotective benefits, opposed to the detrimental effects of the ACE/AII/AT1R pathway [38, 39]. Although a preliminary genetic analysis in neurodegenerative diseases showed no significant expression changes in human subjects [40], some alterations in Ang-(1-7) or ACE2 protein levels in AD and PD have been revealed. Ang-(1-7) plasma levels were decreased in both PD and AD patients compared to the controls [41–43]. Moreover, the plasma concentration of Ang-(1-7) positively correlated with cognitive functions in AD [42]. The analyses on ACE2 showed contradictory results [41, 44, 45], and no studies on Mas levels have been performed. The available data is scarce, thus analysing genetic variability in Ang-(1-7)/ACE2/Mas axis in terms of PD susceptibility, or cognitive decline in PD, could shed some light on the possible neuroprotection exerted by this pathway. Moreover, ACE2 is highly expressed in the substantia nigra and serves as a cellular doorway for the SARS-CoV-2 virus [46]. Some cases of parkinsonism following COVID-19 have been reported [47]. What is the nature of this correlation, as well as whether the SARS-CoV-2 infection may result in a fully developed PD, is still unknown.

We are obliged to indicate some limitations in our study. First of all, the prevalence of dementia in PD increases with age, thus it is possible that in the follow-up, MCI or dementia could affect more participants who were cognitively intact at the baseline. The next step was to assess potential risk factors in patients whose cognitive function had deteriorated. A disadvantage of our study may be the fact that the groups were not matched according to co-morbidities like arterial hypertension, diabetes mellitus, dyslipidaemia, and other vascular risk factors that could influence the diagnosis of cognitive decline.

Another issue may be the distribution of the *ACE* I/D polymorphism in the PD-MCI group, which was not in Hardy–Weinberg equilibrium. This could have resulted from genotyping errors, as the method used (PCR with subsequent electrophoresis) is less precise than real-time PCR. However, the numbers seemed to be equally affected in each of the genotypes, without tending towards any of the homozygotes, thus not indicating an analysis inaccuracy that would probably result in outnumbered DD heterozygotes. Allele and genotype frequencies of *ACE* I/D between the groups did not differ significantly, not correlating with the cognitive phenotype. This deviation could disappear in a larger sample or in a group of participants from more than two centres.

Finally, it is also possible that the genetic variability in the material extracted from peripheral blood may be different in local tissues, i.e. in the brain, thus not reflecting the actual correlation between the polymorphisms in RAS genes and cognitive impairment.

Nevertheless, our study is the first to extensively analyse the genetic variation in RAS with regard to PD, cognitive decline and dementia.

## Conclusions

In the presented analysis we did not find any association between genetic polymorphisms in RAS and mild cognitive impairment or dementia in Parkinson’s disease patients. However, the impact of RAS and the variability in its genes on cognitive decline in PD may be indirect, influencing the risk of cerebral hyperintensities or the impact of antihypertensive treatment with ACE inhibitors. The available research is scarce, not providing any answers to these hypotheses. Further work on the susceptibility factors for dementia in Parkinson’s disease is needed.

## Supplementary Information

Below is the link to the electronic supplementary material.

Supplementary file1 (PDF 103 kb)

## Data Availability

The data that support the findings of this study, except for patients’ identifiers, are available from the corresponding author upon reasonable request.
