# Supplementary material for: Genetic polymorphisms in the renin-angiotensin system and cognitive decline in Parkinson’s disease
Source: Mol Biol Rep. 2021 Jul 23;48(7):5541–8. doi: 10.1007/s11033-021-06569-6 (PMC8301732; doi:10.1007/s11033-021-06569-6)
Supplement: Supplementary file 1 — Supplementary file1 (PDF 103 kb) [file 11033_2021_6569_MOESM1_ESM.pdf]

Supplementary Table 1. Odds ratios (OR) and 95% confidence intervals (95%CI) for the analysed polymorphisms calculated between the groups: PD patients without cognitive impairment (PD-NCI), with mild cognitive impairment (PD-MCI), or with Parkinson's disease dementia (PDD).

Polymorphism	Genotype/allele	PD-NCI vs. PD-MCI+PDD OR (95%CI)	PD-NCI+PD-MCI vs. PDD OR (95%CI)	PD-NCI vs. PD-MCI OR (95%CI)	PD-NCI vs. PDD OR (95%CI)
AGT rs699:T>C	TT	-	-	-	-
	CT	0.78 (0.39-1.57)	0.70 (0.36-1.37)	0.87 (0.41-1.82)	0.64 (0.28-1.47)
	CC	0.59 (0.27-1.30)	0.91 (0.42-1.95)	0.57 (0.24-1.32)	0.64 (0.25-1.62)
	CT+CC	0.71 (0.37-1.37)	0.77 (0.41-1.42)	0.75 (0.38-1.52)	0.64 (0.29-1.39)
AGT rs4762:C>T	CC	-	-	-	-
	CT	0.74 (0.40-1.38)	0.64 (0.32-1.28)	0.84 (0.43-1.63)	0.57 (0.26-1.28)
	TT	0.44 (0.09-2.03)	0.43 (0.05-3.64)	0.52 (0.10-2.70)	0.29 (0.03-2.93)
	CT+TT	0.70 (0.38-1.27)	0.62 (0.32-1.21)	0.80 (0.42-1.51)	0.54 (0.25-1.17)
AGTR1 rs5186:A>C	AA	-	-	-	-
	AC	1.10 (0.61-1.96)	0.84 (0.46-1.53)	1.19 (0.64-2.21)	0.94 (0.46-1.93)
	CC	1.46 (0.45-4.69)	1.28 (0.45-3.63)	1.39 (0.40-4.83)	1.59 (0.41-6.10)
	AC+CC	1.14 (0.65-2.00)	0.90 (0.51-1.59)	1.21 (0.67-2.20)	1.02 (0.52-2.02)
AGTR2 (X chr.) rs5194:A>G	G	-	-	-	-
	A	1.17 (0.73-1.87)	0.91 (0.57-1.45)	1.24 (0.75-2.04)	1.04 (0.59-1.86)
AGTR2 (X chr.) rs5194:A>G only men	G	-	-	-	-
	A	0.97 (0.46-2.01)	0.75 (0.35-1.63)	1.09 (0.49-2.40)	0.79 (0.32-1.95)
AGTR2 (X chr.) rs5194:A>G only women	GG	-	-	-	-
	AG	0.98 (0.31-3.12)	1.02 (0.32-3.26)	0.97 (0.28-3.32)	1.00 (0.24-4.14)
	AA	1.73 (0.43-6.98)	1.04 (0.29-3.80)	1.80 (0.42-7.76)	1.60 (0.30-8.49)
	AG+AA	1.16 (0.38-3.56)	1.03 (0.34-3.14)	1.17 (0.36-3.83)	1.14 (0.29-4.50)
AGTR2 (X chr.) rs1403543:G>A	A	-	-	-	-
	G	0.96 (0.60-1.54)	0.98 (0.61-1.57)	0.97 (0.59-1.60)	0.96 (0.54-1.70)

AGTR2 (X chr.) rs1403543:G>A only men	A	-	-	-	-
	G	1.09 (0.53-2.28)	0.78 (0.36-1.69)	1.24 (0.56-2.73)	0.88 (0.36-2.18)
AGTR2 (X chr.) rs1403543:G>A only women	AA	-	-	-	-
	AG	0.70 (0.22-2.16)	1.24 (0.43-3.63)	0.63 (0.19-2.03)	0.89 (0.22-3.53)
	GG	0.76 (0.20-2.87)	1.29 (0.37-4.52)	0.68 (0.17-2.73)	0.97 (0.19-4.87)
	AG+GG	0.71 (0.24-2.12)	1.26 (0.45-3.51)	0.64 (0.21-1.98)	0.91 (0.24-3.44)
ACE I/D[a]	DD	-	-	-	-
	ID	0.83 (0.36-1.91)	0.97 (0.43-2.18)	0.82 (0.34-1.97)	0.85 (0.31-2.34)
	II	0.64 (0.26-1.61)	1.26 (0.51-3.10)	0.54 (0.20-1.45)	0.85 (0.28-2.58)
	ID+II	0.77 (0.35-1.72)	1.05 (0.48-2.29)	0.73 (0.31-1.70)	0.85 (0.32-2.26)

P-values for alleles and genotypes calculated by means of Fisher exact test in relation to major allele or homozygotes for a major allele. All calculated p-values > 0.05.

[a] - the genotype distribution of *ACE* I/D in PD-MCI group was not in Hardy-Weinberg equilibrium
